# Publisher Correction: Hemozoin induces malaria via activation of DNA damage, p38 MAPK and neurodegenerative pathways in a human iPSC-derived neuronal model of cerebral malaria

**DOI:** 10.1038/s41598-024-80594-w

**Published:** 2024-11-28

**Authors:** Abida lslam Pranty, Leon-Phillip Szepanowski, Wasco Wruck, Akua Afriyie Karikari, James Adjaye

**Affiliations:** 1 Institute for Stem Cell Research and Regenerative Medicine, Medical Faculty, Heinrich-Heine University, 40225 Düsseldorf, Germany; 2 Zayed Centre for Research Into Rare Diseases in Children (ZCR), University College London – EGA Institute for Women’s Health, 20 Guilford Street, WC1N 1DZ London, United Kingdom; 3 Department of Biomedical Sciences, School of Allied Health Sciences, College of Health and Allied Sciences, University of Cape Coast, Cape Coast, Ghana

Correction to: *Scientific Reports* 10.1038/s41598-024-76259-3, published online: 23 October 2024

The original version of this Article contained an error in the spelling of the author Abida Islam Pranty which was incorrectly given as Abida Lslam Pranty.

The original Article has been corrected.

