# Supplementary material for: Hemozoin induces malaria via activation of DNA damage, p38 MAPK and neurodegenerative pathways in a human iPSC-derived neuronal model of cerebral malaria
Source: Sci Rep. 2024 Oct 23;14:24959. doi: 10.1038/s41598-024-76259-3 (PMC11496667; doi:10.1038/s41598-024-76259-3)
Supplement: Supplementary file 7 — Supplementary Material 7 [file 41598_2024_76259_MOESM7_ESM.pdf]

Supplementary Fig. 1:

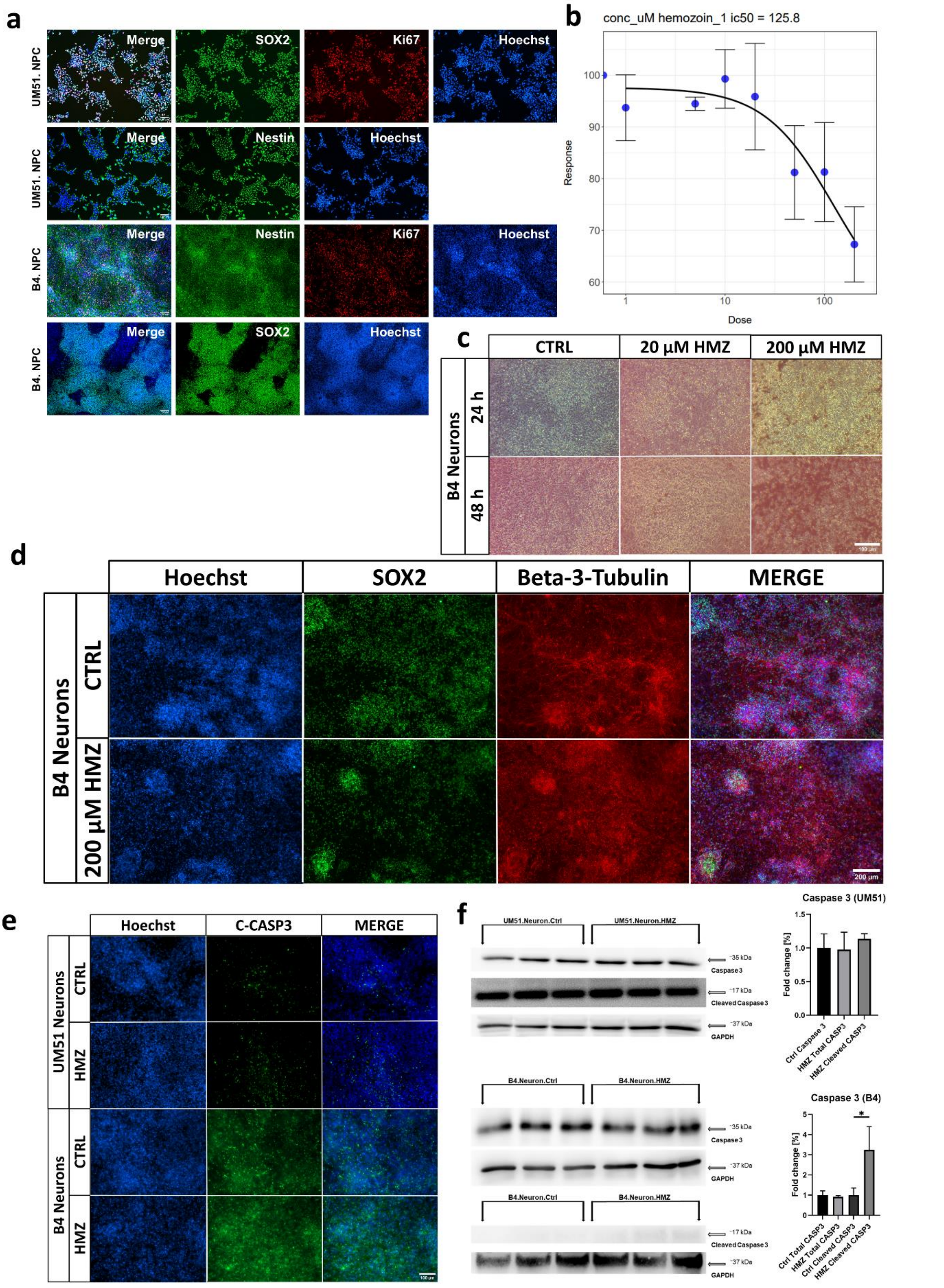

Supplementary Figure 1. Preliminary and optimal-dose experiments for HMZ treatments (a) Representative ICC images of Nestin-, SOX2- and KI67-positive cells in cultured neural progenitor cells**.** **(b)** Dose-response curve of B4-derived neuronal cultures treated with 0, 1, 5, 10, 20, 50, 100 and 200

µM HMZ. **(c)** Representative brightfield images of control, 20 µM and 200 µM HMZ-treated B4 iPSC-derived neuronal cultures at 24h and 48h of exposure. Scale bar 100 µm. **(d)** Representative ICC images of SOX2- and β3-Tubulin-positive cells in B4-derived neuronal networks after 48h of 200 µM HMZ exposure in comparison to control. Scale bar 200 µm. **(e)** Representative ICC images of Cleaved Caspase 3-positive cells in UM51- and B4-derived neuronal cultures after 48h exposure to 20 µM HMZ in comparison to control. Scale bar 100 µm. **(f)** WB analyses and quantification of WB analyses for total Caspase 3 and Cleaved Caspase 3 in in UM51- and B4-derived neuronal cultures after 48h HMZ exposure in comparison to control. n=3 for each condition, blots depict mean and error bars depict SD of all experiments. Asterisk (*) depicts significance, which is indicated by *p<0.05.

Supplementary Fig. 2:

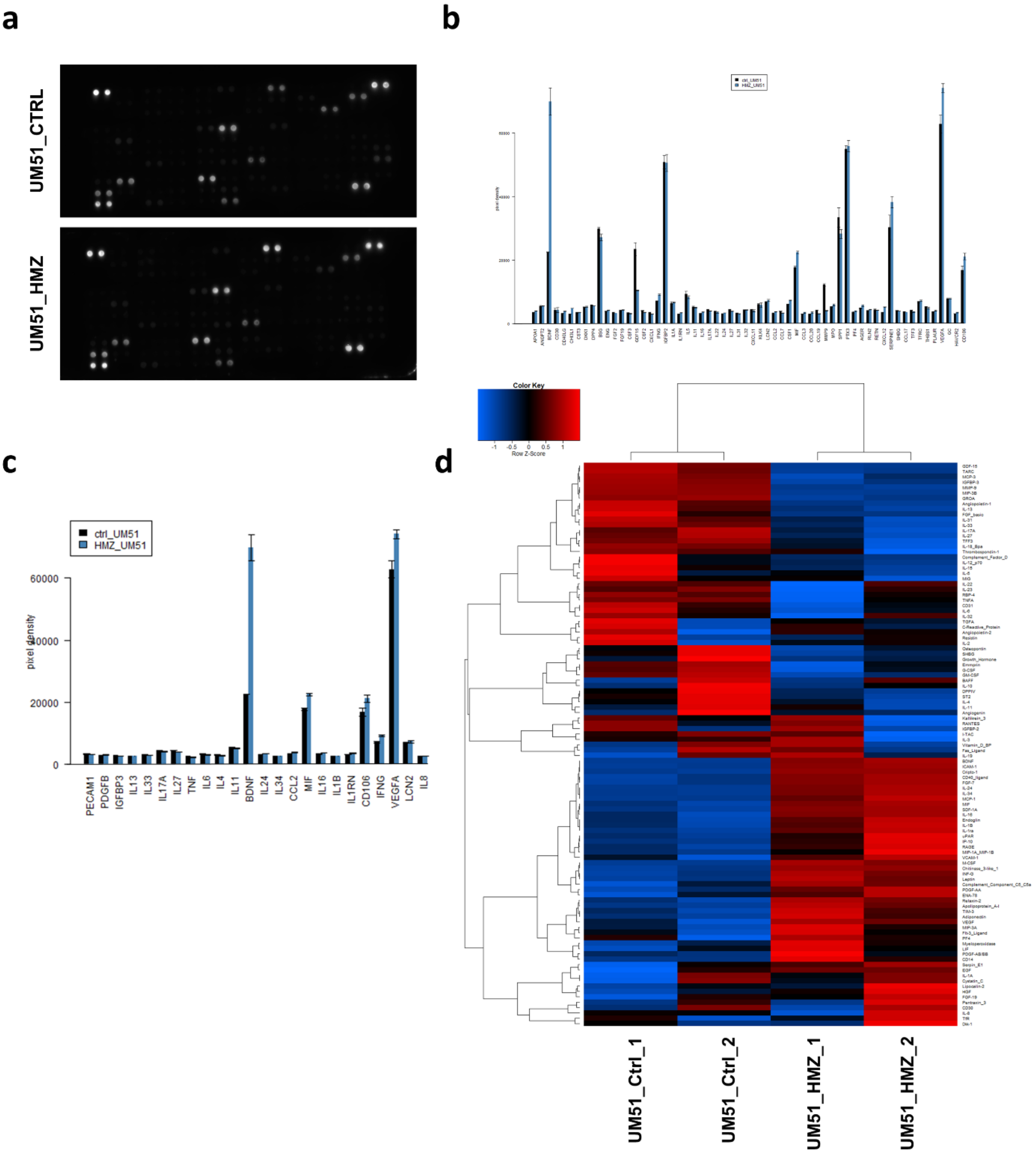

Supplementary Figure 2. UM51 neuronal culture cytokine array. (a) Cytokine array blots utilized for detecting altered secretomes in UM51 neuronal cultures after 48h 20 µM HMZ exposure in comparison to control. **(b)** Multibar plot showing all chemo- and cytokines with pixel density values over background

pixel density. **(c)** Multibar plot for selected chemo- and cytokines showcased as heatmap in Figure 2a. **(d)** Heatmap using Pearson correlation as similarity measure depicting all examined chemo- and cytokines. Error bars denote the standard error of the mean (b,c).

Supplementary Fig. 3:

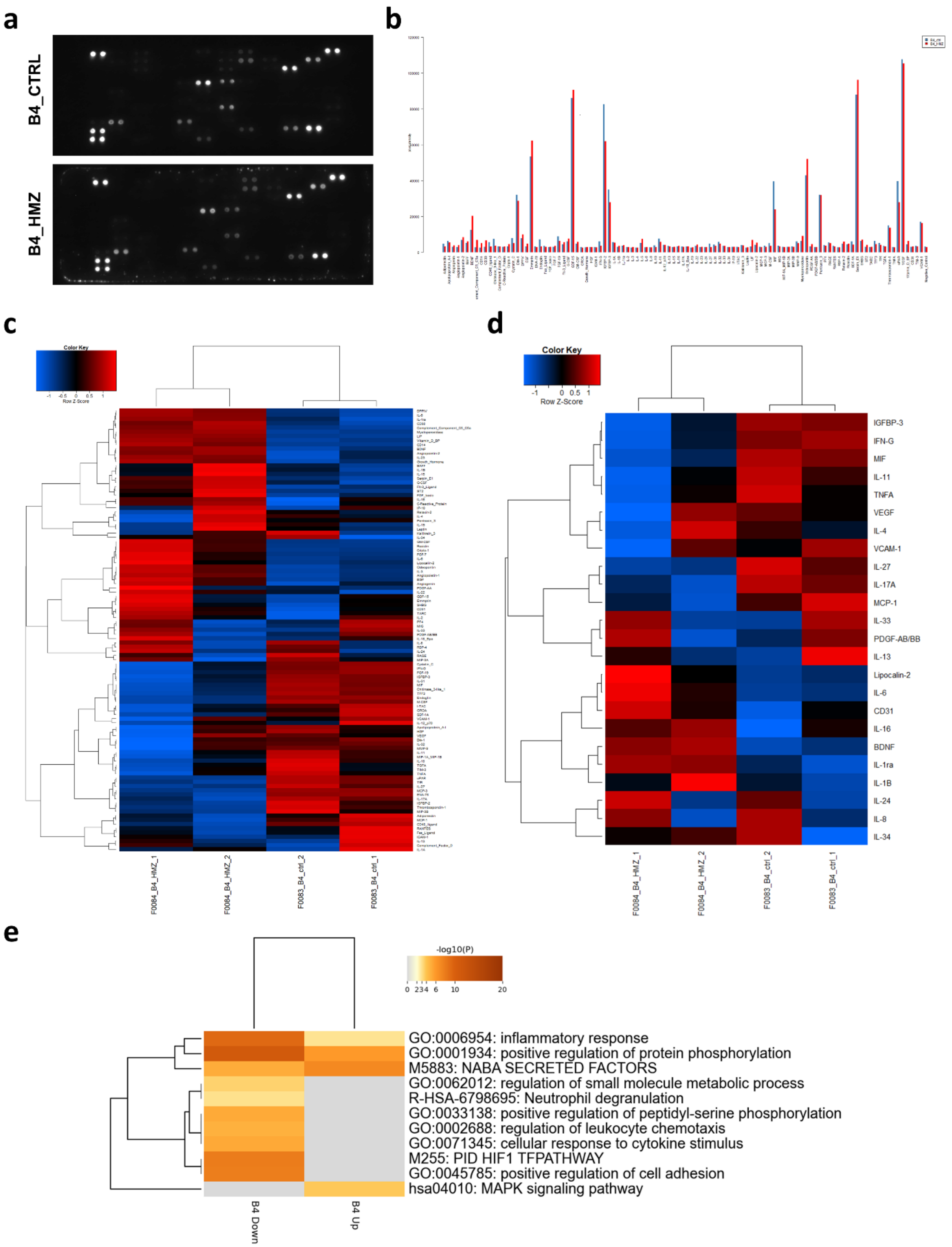

Supplementary Figure 3. Cytokine array of B4 neuronal cultures. (a) Cytokine array blots utilized for detecting altered secretomes in B4 neuronal cultures after 48h 20 µM HMZ exposure in comparison to control. **(b)** Multibar plot showing all chemo- and cytokines with pixel density values above background pixel density. **(c)** Pearson´s heatmap depicting all examined chemo- and cytokines. **(d)** Pearson´s heatmap depicting selected chemo- and cytokines regulated in B4 neuronal cultures after 48 h HMZ exposure in comparison to control. **(e)** Metascape-generated heatmap comparing the sets of up- and downregulated chemo- and cytokines derived from B4 neuronal cultures after 48 h HMZ exposure, revealing a secretome signature involved in i.a. inflammatory response and MAPK signalling pathway. Error bars denote the standard error of the mean (b).

Supplementary Fig. 4:

a

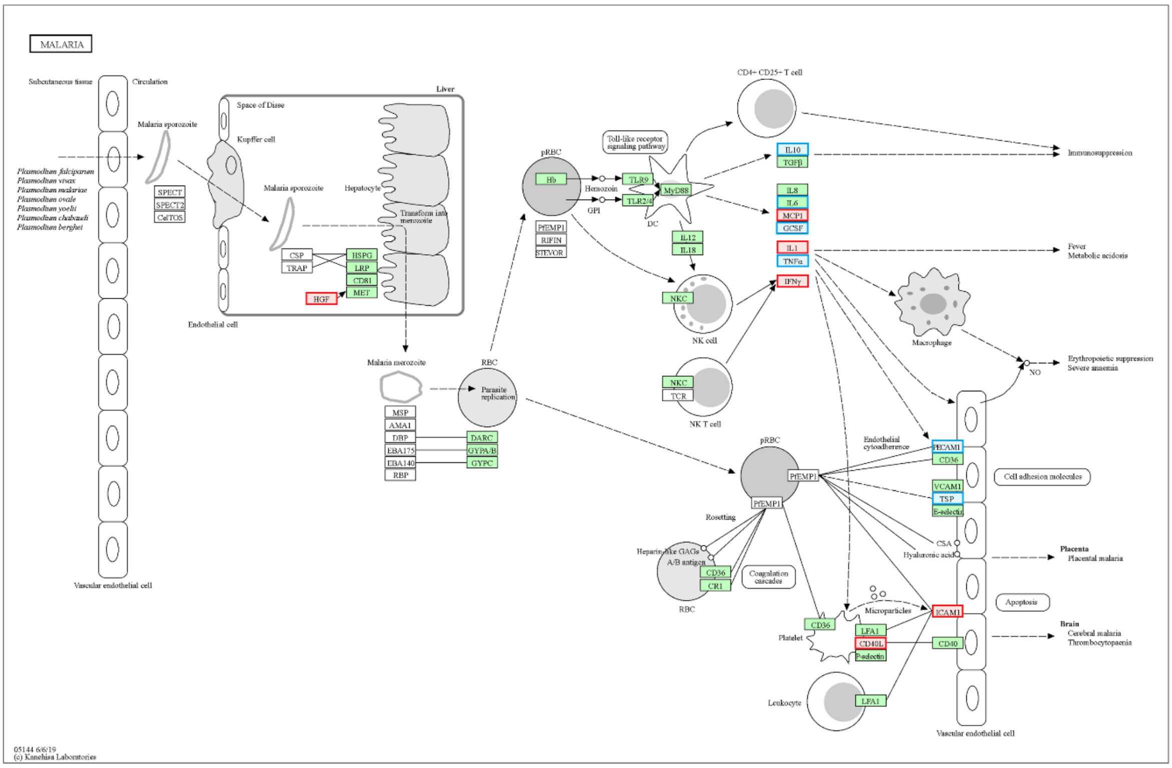

b

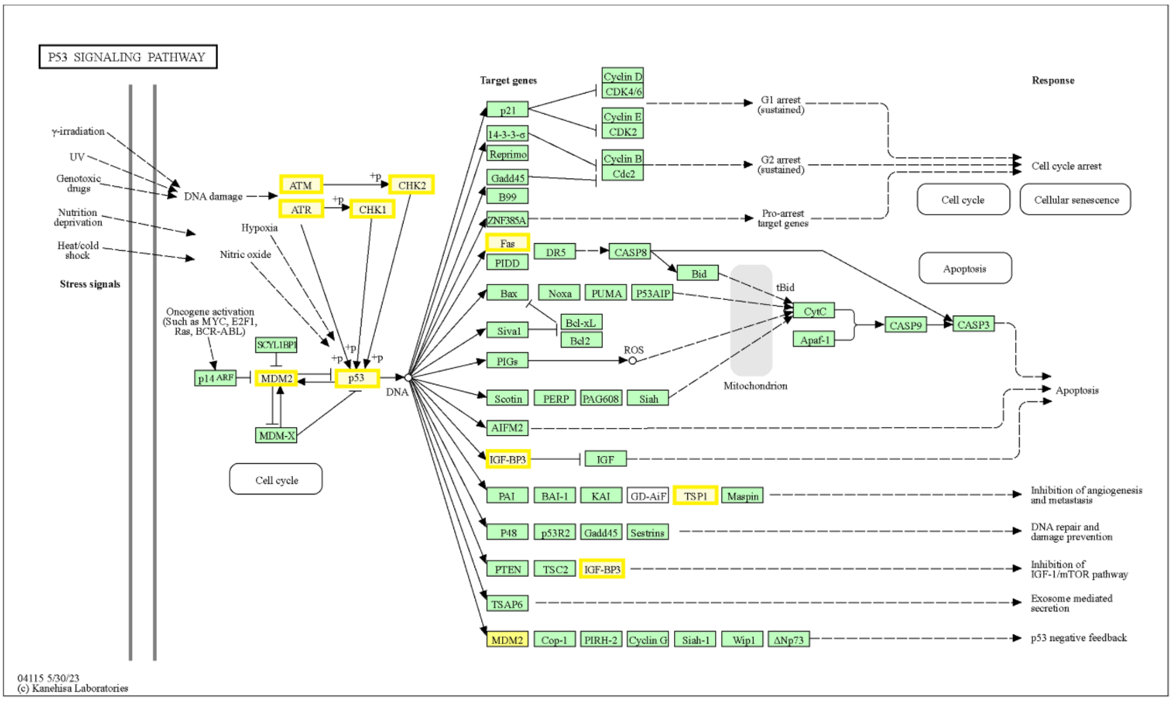

Supplementary Figure 4. Analysis of the KEGG pathways - Malaria and p53 signalling pathways in iPSC-derived neuronal cultures. (a) Schematic of the KEGG pathway Malaria. Cytokines upregulated in the secretome of UM51 neuronal cultures are marked in red, downregulated in blue. **(b)** Schematic of the KEGG pathway p53 signalling pathway. Regulated genes are highlighted in yellow, and non-regulated in grey.

Supplementary Fig. 5:

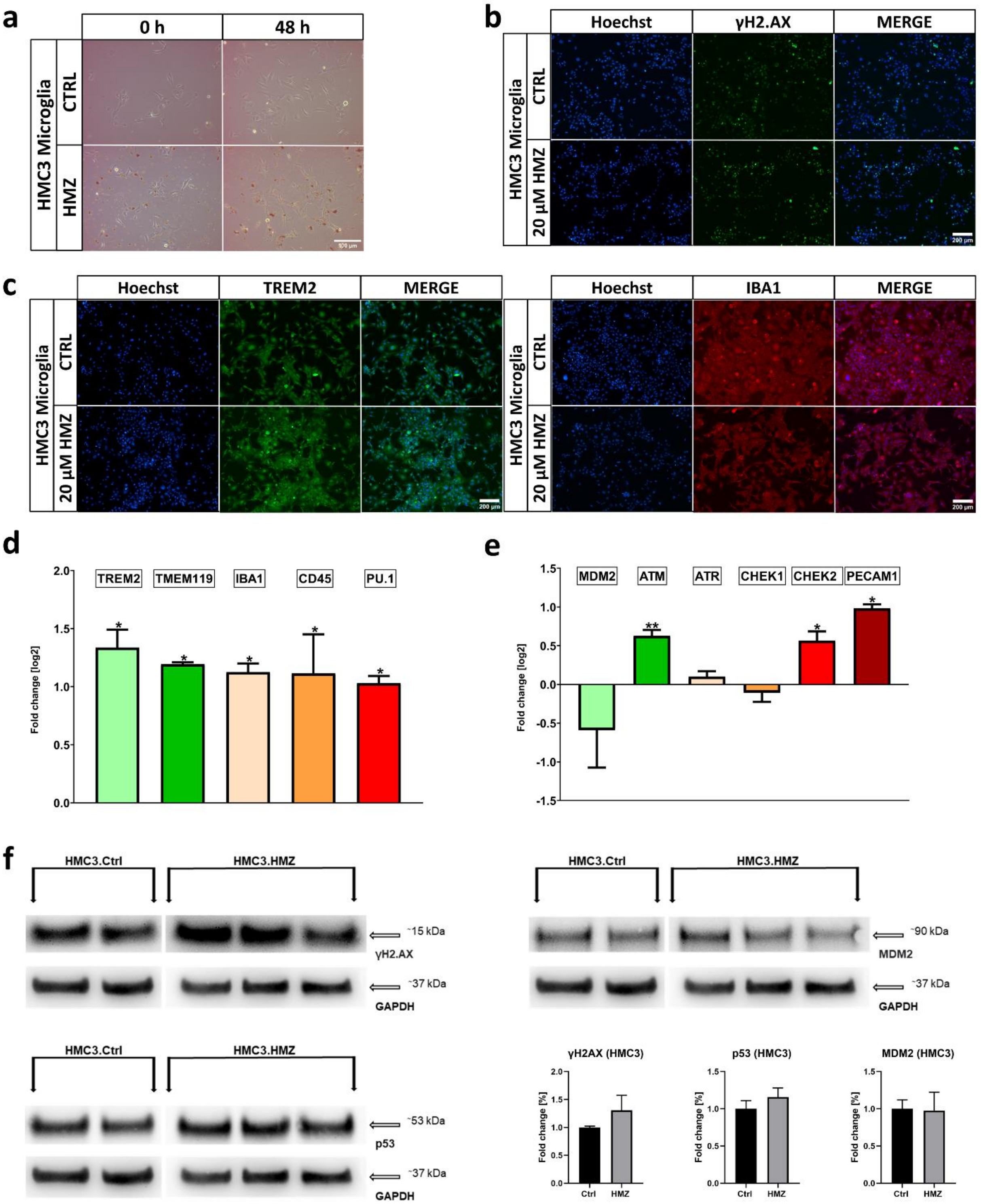

Supplementary Figure 5. HMC3 cells show signs of microglial activation and activation of DNA damage response. (a) Representative bright field images of control and 20 µM HMZ-treated HMC3 cells at 0h and 48h of exposure. Scale bar 100 µm. **(b,c)** Representative ICC images of γH2AX-, TREM2- and IBA1-positive HMC3 cells after 48h HMZ exposure in comparison to control. Scale bar 200 µm. **(d)** Relative mRNA expression analysis of *TREM2, TMEM119, IBA1, CD45* and *PU.1* in HMC3 cells after 48h HMZ exposure in comparison to control. **(e)** Relative mRNA expression analysis of *MDM2, ATM, ATR, CHEK1, CHEK2* and *PECAM1* in HMC3 cells after 48h HMZ exposure in comparison to control. **(f)** WB analyses and quantification of WB analyses for γH2AX, MDM2 and p53 after 48h HMZ exposure in comparison to control. Values were normalized to GAPDH and subsequently to control

samples. Ctrl n=2; HMZ n=3. (d,e) n=3; blots depict mean and error bars depict SD of all experiments. Asterisk (*) depicts significance, which is indicated by *$p<0.05$; **$p<0.01$.
